# Hydrolyzed Polyacrylamide as an In Situ Assistant in the Nucleation and Growth of Gold Nanoparticles

**DOI:** 10.3390/ma15238557

**Published:** 2022-12-01

**Authors:** Nery M. Aguilar, José Manuel Pérez-Aguilar, Valeria J. González-Coronel, Hugo Martínez-Gutiérrez, Teresa Zayas Pérez, Guillermo Soriano-Moro, Brenda L. Sánchez-Gaytán

**Affiliations:** 1 Chemistry Center, Science Institute, Meritorious Autonomous University of Puebla (BUAP), University City, Puebla 72570, Mexico; 2 School of Chemical Sciences, Meritorious Autonomous University of Puebla (BUAP), University City, Puebla 72570, Mexico; 3 School of Chemical Engineering, Meritorious Autonomous University of Puebla (BUAP), University City, Puebla 72570, Mexico; 4 National Polytechnic Institute (IPN), Center for Nanosciences and Micro and Nanotechnologies, Luis Enrique Erro, Mexico City 07738, Mexico

**Keywords:** hydrolyzed polyacrylamide, nanocomposites, gold nanoparticles

## Abstract

The modulation of nanoparticles’ size, shape, and dispersion by polymers has attracted particular attention in different fields. Nevertheless, there is a lack of information regarding the use of charged macromolecules as assistants in the nanostructures’ nucleation and growth processes. Prompted by this, the in situ synthesis of gold nanoparticles (AuNPs) aided by hydrolyzed polyacrylamides (HPAM), with different chemical structures, was developed. In contrast to the conventional synthesis of nanostructures assisted by polyacrylamide, here, the polymerization, hydrolysis, and nanostructure formation processes were carried out simultaneously in the same milieu. Likewise, the growing chains acted as a template for the nanoparticles’ growth, so their conformations and chemical structure, especially the amount of charges along the chain, played an important role in the AuNPs’ morphology, size, and some of the final composite features. The nanocomposite was thoroughly characterized with appropriate techniques, including ATR–FTIR, GPC, UV–Vis, and SEM.

## 1. Introduction

Hydrolyzed polyacrylamide (HPAM) is one of the most studied polyacrylamide (PAM) derivatives due to its lateral anionic carboxylate groups, which strongly impact its solubility, stability, and structural conformation in solution [1,2]. Usually, this macromolecule is prepared by PAM hydrolysis, where the pH conditions can be modulated to produce a completely or a partially hydrolyzed structure [3]. Recently, HPAM has been used in the design of novel composite materials, since its chemical functionality allows favorable interactions with other molecules or nanostructures. For instance, it has been shown that HPAM is frequently grafted with some inorganic nanoparticles, such as SiO_2_, TiO_2_, and Al_2_O_3_ [4,5]. The grafting process of the polymer and nanostructures can be performed either through polymerization on the surface of the previously synthesized nanoparticles or by chemical interactions between nanoparticles’ surfaces and HPAM [6]. The resulting nanocomposites tend to display new or enhanced mechanical, rheological, and thermal features that are frequently applied in oil recovery applications and in the design of energy storage devices [7,8,9]. Nevertheless, to the best of our knowledge, there is no relevant information regarding the use of HPAM as a modulator in metallic nanoparticle formation, despite its chemical and structural advantages; the charged functional groups of the hydrolyzed segments could favor an interaction with the reducing metal atoms and promote the nanoparticles’ nucleation–growth process to occur along the chains. Furthermore, depending on the chemical environment, HPAM tends to adopt different conformations and assemblies in solution [10], which can strongly influence the nanoparticles’ size, morphology, and dispersion.

Herein, hydrolyzed polyacrylamides prepared at different pH conditions are used as assistants in gold nanoparticles’ (AuNPs) formation by using inverse emulsion polymerization, which usually yields high-molecular-weight polyacrylamides [11,12]. Importantly, in our approach, the polymerization, hydrolysis, and growth of the nanostructures occurred simultaneously. The relevance of carrying out this in situ method is that the acrylamide monomer promotes the gold ions’ reduction [13,14]. Additionally, the different pH values tested for the hydrolysis process (5, 7, and 14) yield macromolecules with different proportions of hydrolyzed segments. Therefore, each of these polymeric structures generates a different chemical environment that will affect the nanoparticles’ growth process. Thus, nanoparticles with different size, morphology, and dispersion are obtained and the different results can be easily visualized due to the size- and shape-dependent optical properties of AuNPs [15,16,17]. 

## 2. Materials and Methods

### 2.1. Materials

Acrylamide monomer (AM; ≥99%), 4,4′-azobis(4-cyanovaleric acid) (ACVA; ≥98%), and Gold(III) chloride solution (HAuCl_4_; ≥99.9%) were purchased from Sigma-Aldrich. Sodium hydroxide (NaOH; 98.6%) was from J. T. Baker. Soy lecithin and toluene (99.5%) were from Química Mercurio, Puebla Mexico. All materials were used without further purification. 

### 2.2. Synthesis of HPAM and Growth of Gold Nanoparticles by Varying the pH of the Reaction 

The continuous phase of the synthesis process was prepared by dissolving soy lecithin (81 mg) in toluene (10 mL). The dispersed phase was prepared by dissolving AM (400 mg) in the gold precursor solution (8 mL, 0.5 mM HAuCl_4_). 

The hydrolysis process was carried out by adjusting the pH of the dispersed phase to 5, 7, and 14. The gold precursor solution possessed a pH of 5, so no adjustment was necessary for the acidic pH (HPAM-Au5). For pH 7, a small amount of NaOH was added (HPAM-Au7). Basic pH adjustment was achieved by adding 100 mg of NaOH to the solution (HPAM-Au14).

After the homogenization of both phases was performed under continuous stirring, the system was purged with a flow of nitrogen only for 5 min, sealed, and heated. When the temperature reached 80 °C, ACVA (16 mg dissolved in 2 mL of toluene) was added to the reaction system. The reactions were carried out for 105 min.

The final product was purified by concentrating the dispersed phase and precipitating it with methanol. Finally, the materials were dried at 60 °C. 

### 2.3. Characterization

Attenuated Total Reflectance–Fourier Transform Infrared Spectroscopy (ATR–FTIR) measurements were performed in a Cary 630 spectrometer (Agilent Technologies, Santa Clara, CA, USA) in a range from 900 to 4000 cm^−1^, using a universal attenuated total reflectance (ATR) accessory.

Gel permeation chromatography (GPC) was carried out with a sample concentration of 1 g/L using an HPLC instrument (Agilent Technologies, Santa Clara, CA, USA), model 1260 Infinity, coupled with ultraviolet and refractive index detectors using a series of AGUA-GEL-OH (30, 40 and 50 nm) columns. A buffer solution composed of NaNO_3_ (0.2 M), NaH_2_PO_4_ (0.01 M), NaN_3_ (100 ppm) was used as the mobile phase at a flow rate 0.5 mL/min. Prior to injection, the nanocomposites were centrifuged 4 times, at intervals of 30 min, using a spectrafuge 24D microcentrifuge (Labnet, Big Flats, NY, USA) to eliminate the nanoparticles.

Scanning transmission electron microscopy (STEM) micrographs were acquired with 30 kV using a JSM-7800F scanning electron microscope (JEOL Ltd., Tokyo, Japan). 

UV–Vis spectroscopy measurements were carried out on a Varian Cary 50 spectrophotometer (Agilent Technologies) with a xenon lamp. Measurements were made of nanocomposites aqueous solutions using a quartz cuvette of 1 cm path length.

Nanoparticle size was monitored by Dynamic Light Scattering (DLS) using a ZEN3690 zetasizer, Nano ZS90 (Malvern Panalytical, Malvern, UK). The distributions obtained included both the hydrolyzed polyacrylamide and the gold nanoparticles. 

The steady shear viscosity measurements were obtained in a rotational Rheolab QC rheometer (Anton-Paar) using a geometry double-walled cylinder (DG42). Measurements were made of nanocomposites’ aqueous solutions with a concentration of 23.4 mg/mL at room temperature.

## 3. Results and Discussion

Inverse emulsion polymerization, as well as emulsion polymerization, are processes that take place in a biphasic system comprising a continuous phase that solubilizes the initiator and a monomer-containing dispersed phase located within micelles called monomer droplets. 

This process has three characteristic stages [18]; in the first stage, the migration of monomers from the monomer droplets to the continuous phase occurs, followed by the formation of small monomer-containing micelles. During stage two, there is a subsequent increase in the size of these aforementioned micelles, driven by the polymer chain growth. Lastly, in the final stage, the termination process takes place. 

**Figure 1:**
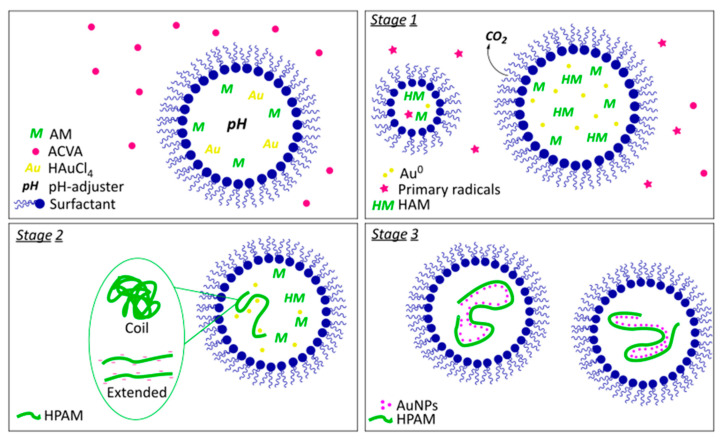
Representation of the reaction process stages. Stage 1: hydrolysis of some monomers. Oxidation of hydrolyzed monomers to CO_2_ and gold reduction. Initiation of polymerization followed by the propagation step. Stage 2: Growth of nanoparticles in the polymeric template (coil conformation at pH of 5 and 7, extended conformation at pH of 14). Stage 3: nanocomposite formation.

In this work, however, these stages are slightly modified because the polymerization, hydrolysis, and nanoparticle formation processes happen simultaneously in the dispersed phase (Figure 1). 

During stage one, the polymerization process begins with the generation of primary radicals that subsequently activate the monomers that have migrated from the monomer droplet into the small micelles (i.e., initiation step). Then, the chains begin to grow (i.e., the propagation step), which increases the size of these micelles. Nevertheless, compared to typical acrylamide polymerization, this step is less favorable since some of these monomers are oxidized to CO_2_ by the reduction of the gold ions [19], while some are hydrolyzed due to the pH conditions. Thus, there is a synthetic tug-of-war between the reduction and hydrolysis reactions and the chain growth. Importantly, at pH 5 and 7, the characteristic purple color, indicative of the AuNPs’ formation, appears 3 min after the beginning of the reaction. At pH 14, a reddish color appears 40 min after the beginning of the reaction, which may suggest that the AM hydrolysis process at this pH is more favored than the gold reduction relative to the other pH values investigated.

In stage two, the polymer chains continued to grow, and we suggest that the nucleation/growth of gold nanoparticles occurred along these, so the polymer chains’ conformations can significantly impact the nanoparticles’ dispersion, size, and shape. Chains with few hydrolyzed segments favored coiled conformations, similar to those exhibited by the polyacrylamide in solution, which generates irregular areas where nucleation and growth can take place. The presence of more charged segments in the highly hydrolyzed chains, on the other hand, generates electrostatic repulsions that can force the chains to remain extended and favors interactions with water molecules, restricting the chains’ movement. Thus, these conformations provide homogeneous zones where nanoparticles can nucleate and grow.

Finally, in stage 3, the entire process was completed, resulting in the final nanocomposites.

### 3.1. Attenuated Total Reflectance–Fourier Transform Infrared Spectroscopy (ATR–FTIR)

**Figure 2:**
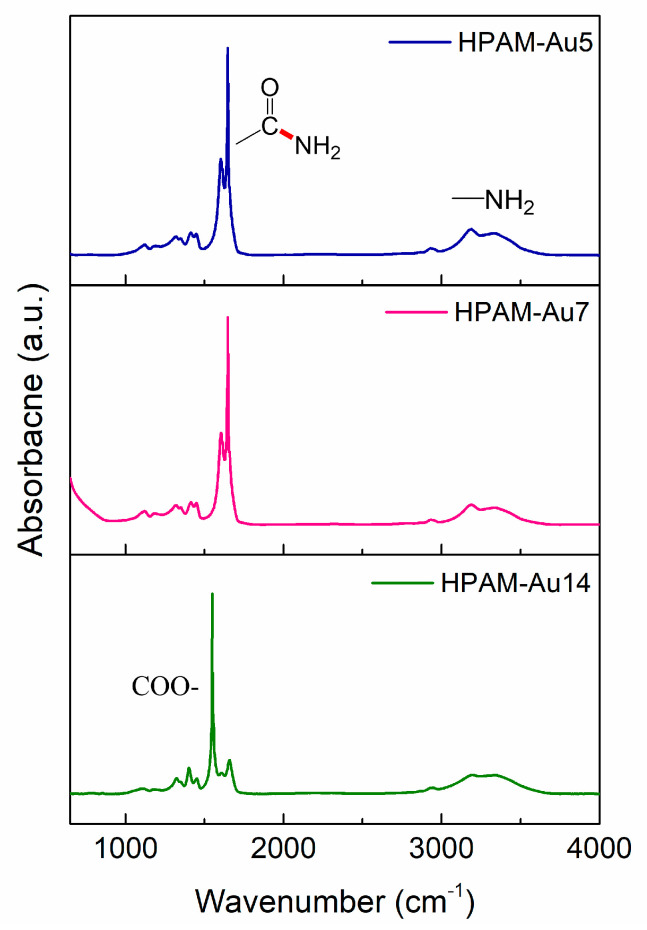
ATR–FTIR spectra comparison between nanocomposites prepared at different pHs: HPAM–Au5 (acidic), HPAM–Au7 (neutral), HPAM–Au14 (basic).

ATR–FTIR was employed to visualize the structural variations of HPAMs prepared at different pHs (Figure 2). The spectra obtained for HPAM-Au5 and HPAM-Au7 are similar. In both cases, the bands indicative of the primary amine (-NH_2_) appear at 3400 and 3200 cm^−1^. At 2980 cm^−1^, the band corresponding to the CH_2_ asymmetric vibration of the polymer backbone can be seen. The C=O stretching vibration bands of the amide group are observed at 1645 cm^−1^ and 1603 cm^−1^. This indicates that these polymers have mostly non-hydrolyzed segments and an analogue chemical structure. Thus, it is expected that the impact of these macromolecules in nanoparticle formation will be similar.

Meanwhile, the HPAM-Au14 spectrum displays less defined vibration bands of -NH_2_. Moreover, the C=O stretching vibration bands of amides are replaced by the stretching modes of the carboxylate group (COO^−^) at 1659 and 1550 cm^−1^, evidencing that most of its structure is hydrolyzed [20]. 

### 3.2. Gel Permeation Chromatography (GPC)

The number- and weight-average molecular weight values (M_n_ and M_w_, respectively) of the different HPAMs that constitute the nanocomposites are within the typical ranges obtained by the inverse emulsion technique, as discussed previously [11,12] (Table 1).

**Figure 3:**
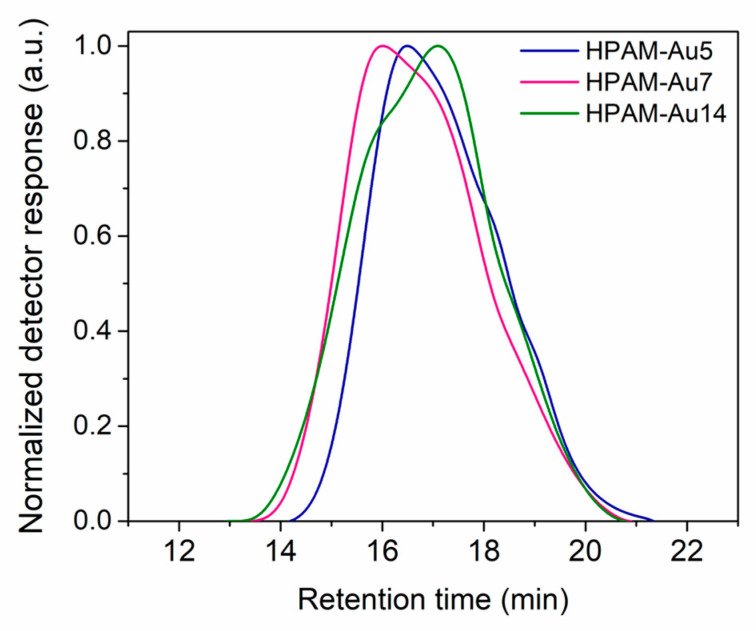
Molecular weight distribution by GPC of the HPAMs obtained at different pHs used to prepare the nanocomposites: HPAM-Au5 (acidic), HPAM-Au7 (neutral), HPAM-Au14 (basic).

Nevertheless, even though high molecular weights are obtained, both the molecular size and the homogeneity of the chains are impacted by the simultaneous reactions (Figure 3). All the molecular weight distributions of HPAMs prepared at different pHs are wide, suggesting the presence of polymer chains with irregular lengths due to the competition between the polymerization, hydrolysis, and monomer oxidation processes, all occurring during the propagation step. Additionally, two populations of different molecular weights can be observed, verifying the presence of hydrolyzed segments in the chains.

### 3.3. UV–Vis and Dynamic Light Scattering (DLS) 

**Figure 4:**
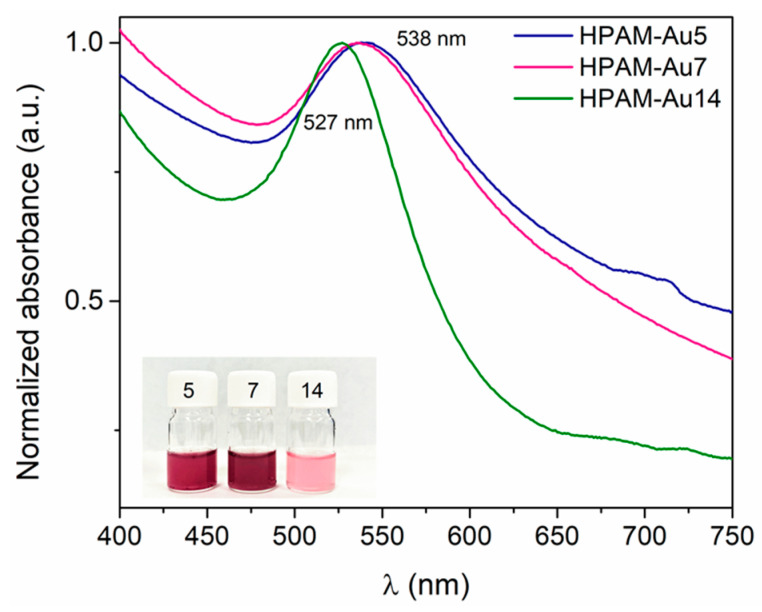
UV–Vis spectra of nanocomposites in aqueous solution: HPAM-Au5 (acidic), HPAM-Au7 (neutral), HPAM-Au14 (basic).

The optical properties displayed by these materials are the result of the influence of HPAMs on the nanoparticles’ formation. The UV–Vis spectra (Figure 4) show that the three nanocomposites present only one plasmon band, characteristic of a spherical morphology. HPAM-Au5 and HPAM-Au7 have a similar wide plasmon with a maximum at 538 nm, suggesting the presence of populations of larger nanoparticles and agglomerates. This implies that HPAMs prepared at these conditions promoted coiled conformations, due to the low amount of charges in their chains, generating irregular areas for the formation of nanoparticles.

HPAM-Au14, on the other hand, has a narrower and defined plasmon with a maximum of 527 nm, typical of nanoparticles with a size of around 20 nm [21,22]. The shape and maximum value of this plasmon imply that the nanospheres are similar in size and shape. This suggests that at highly basic conditions, the considerable number of hydrolyzed segments favors an extended conformation, homogenizing the areas where nanostructures grow. Moreover, the agglomerations are poorly favored due to electrostatic repulsions caused by the large number of hydrolyzed segments in the chains. 

**Figure 5:**
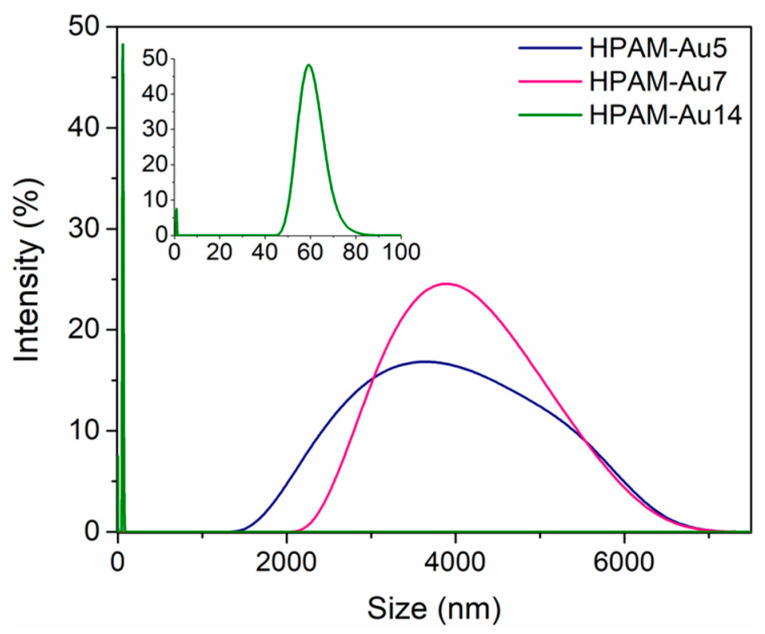
Intensity distributions obtained by DLS of nanocomposites in aqueous solution: HPAM-Au5 (acidic), HPAM-Au7 (neutral), HPAM-Au14 (basic).

In addition to influencing the formation of the nanoparticles, the chemical structure of the formed HPAMs affects the behavior of the nanocomposites in solution (Figure 5). DLS analysis shows dramatic changes in size that depend on the conformations that the different structures tend to adopt. 

The DLS size distribution at acidic and neutral pH suggests nanoparticle/polymer agglomerates because of the favored coil conformation. In contrast, the distribution at basic pH is indicative of the good detection of individual nanoparticles, implying a possible structural arrangement between HPAMs and AuNPs due to the extended conformation generated by the presence of negative charges.

### 3.4. Scanning Transmission Electron Microscopy (STEM) 

**Figure 6:**
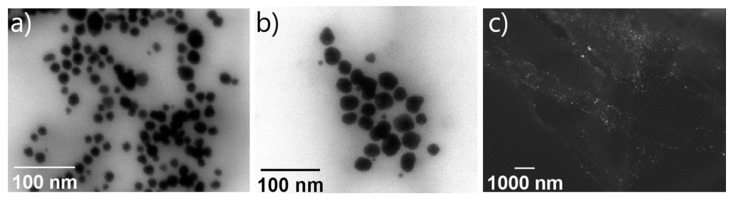
STEM micrographs of nanocomposites: HPAM-Au5 (**a**), HPAM-Au7 (**b**), HPAM-Au14 (**c**).

STEM micrographs (Figure 6) indicate that the morphologies of nanoparticles are mostly spherical, and their dispersion and size were affected by the conditions of the reaction. 

HPAM-Au5 and HPAM-Au7 are composed of heterogeneous nanoparticles and large agglomerates. This is attributed to the irregular nucleation–growth zones due to the coil conformation favored by the polymers with mostly non-hydrolyzed segments.

Meanwhile, as discussed above, HPAM-Au14 has monodisperse nanostructures due to the homogeneous growth areas created by the extended conformation of the HPAMs prepared at basic pH. Likewise, it can be seen that the AuNPs are distributed homogeneously in an arrangement along the polymer, representative of metallo-polyelectrolyte nanocomposites [23].

### 3.5. Steady Shear Viscosity Determination

**Figure 7:**
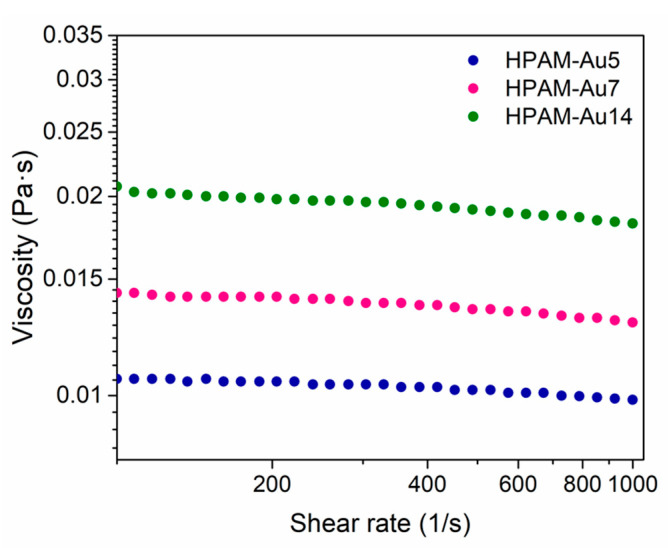
Viscosity variation as a function of the shear rate at 25 °C of nanocomposites in solution prepared at different pHs: HPAM-Au5 (acidic), HPAM-Au7 (neutral), HPAM-Au14 (basic).

The particular steady shear viscosities of these three nanocomposites in solution, predominantly Newtonian (Figure 7), are a consequence of the chemical structure of the HPAMs as well as the size, morphology, and dispersion of the AuNPs. Moreover, this rheological behavior is induced by the arrangement formed by both components, the HPAM and AuNPs, that can favor or not some interactions with the solvent. 

The HPAM-Au5 and HPAM-Au7 nanocomposites show low viscosity since the coil conformation is favored by the non-hydrolyzed segments in the chains. This conformation maintained the monopolar surface nature characteristic of polyacrylamide, which allowed these materials to flow easily [24].

HPAM-Au14, on the other hand, possesses an extended conformation and a unique topology, where nanostructures are found along the polyelectrolyte side. This arrangement favors polymer–nanostructure–solvent interactions by limiting the fluidity of the nanocomposite solution, which increases the viscosity. Indeed, this behavior can be compared to copolymers with hydrophobic segments that show either telechelic, multisticker, or combined associations that affect the viscosity similarly [25].

## 4. Conclusions

We report the novel in situ synthesis by inverse emulsion polymerization of HPAM–AuNP nanocomposites, where the hydrolysis, polymerization, and nanoparticle formation were carried out simultaneously. The results, supported by different characterization techniques, show that all the reactions, i.e., polymerization, hydrolysis, and nanoparticle growth, occur simultaneously. 

In addition, by varying the amount of carboxylate groups in the HPAM structure, some polyelectrolyte nanocomposites’ properties, including optical and rheological, can be affected. At pH 5 and 7, viscous HPAM nanocomposites with a plasmon maximum at 538 nm were obtained. At a basic pH (14), on the other hand, highly viscous HPAM nanocomposites with a plasmon maximum at 527 nm were yielded. The versatility shown by these synthetic approaches, and the nanocomposites’ features, make this work a relevant contribution in the design of new materials.

## Figures and Tables

**Table 1:** Molecular weight values of nanocomposites.

Sample	M_n_ (g/mol)	M_w_ (g/mol)
HPAM-Au5	48,000	207,000
HPAM-Au7	67,000	402,000
HPAM-Au14	61,200	415,000

## Data Availability

The data presented in this study are available from the corresponding authors upon reasonable request.
